# Frequency of body focused repetitive behaviors and comparison to self-injurious behaviors in patients with tic disorders

**DOI:** 10.1038/s41598-025-12023-5

**Published:** 2025-08-25

**Authors:** Natalia Szejko, Heike grosse Schlarmann, Kirsten R. Müller-Vahl

**Affiliations:** 1 Clinic of Psychiatry, Social Psychiatry and Psychotherapy, Hannover Medical School, Carl-Neuberg-Str. 1, 30625 Hannover, Germany; 2 Department of Bioethics, Medical University of Warsaw, Warsaw, Poland

**Keywords:** Tics, Self-injurious behaviors, Body focused repetitive behaviors, Obsessive-compulsive disorder, Attention-deficit/hyperactivity disorder, Quality of life, Movement disorders, Psychiatric disorders

## Abstract

The majority of patients with chronic tic disorders suffer from psychiatric comorbidities, most frequently attention-deficit/hyperactivity disorder (ADHD), obsessive-compulsive disorder (OCD), depression, and anxiety. While characteristics of these psychiatric disorders are widely known, other comorbidities including self-injurious behaviors (SIB) and body focused repetitive behaviors (BFRB) are still under-investigated. Currently, there is a controversial discussion whether BFRB and SIB belong to the same spectrum because of clinical similarities or they represent distinct entities. In this study, we investigated the clinical profile of BFRB in a large sample of adult patients (*N* = 123) with chronic tic disorders. Current *urge* and/or *behavior* to perform BFRB was reported by 59 participants (48%). BFRB were overall more frequent in females, individuals with comorbid OCD, and more severe tics. Both urges and behaviors to perform BFRB had a negative impact on quality of life. When comparing patients with comorbid SIB and those with comorbid BFRB, SIB was found in younger patients with more severe tics and comorbid ADHD. From our data it is suggested that SIB and BFRB belong to the same spectrum with SIB indicating the more severe clinical manifestation.

**Supplementary Information:**

The online version contains supplementary material available at 10.1038/s41598-025-12023-5.

## Introduction

Primary tic disorders including provisional tic disorder (PTD) are characterized by the presence of tics for a period shorter than 12 months and are found in up to 20% of children^1^. In contrast, other types of tic disorders such as chronic motor (CMT) and vocal tic disorders (CVT), and Tourette syndrome (TS) belong to a disease spectrum with TS constitute the most complex and severe phenomenological picture^2,3^. About 80–90% of patients with TS suffer from co-existing psychiatric comorbidities such as attention deficit/hyperactivity disorder (ADHD), obsessive-compulsive disorder (OCD), depression, anxiety, self-injurious behavior (SIB), and body focused repetitive behavior (BFRB). While SIB is a well-known comorbidity in TS, BFRB has been investigated only rarely in this group of patients, despite its clinical overlap and similarity to SIB^4^. This study, therefore, focuses on the clinical phenomenology and correlations of BFRB in patients with tics and its differentiation with SIB. Since BFRB is not well known, neither clearly defined, and its concept is controversially discussed, we first provide current knowledge on BFRB in general, thereafter discuss its main differential diagnoses, including SIB and stereotypes, and finally summarize current knowledge on BFRB in patients with tics.

### BFRB: current concepts and classification

BFRB is defined as an urge to manipulate a part of the body in a harmful way^7^ and is considered to be a very frequent phenomenon. According to recent epidemiological studies, the vast majority of the population engage in such behaviors during their lifetime^5^. The most frequent examples include nail-biting, skin picking, trichotillomania, cheek biting, finger cracking, or mucus fishing syndrome^6^. Although as a whole group, BFRB has not been included neither in the Diagnostic and Statistical Manual of Mental Disorders (DSM-5)^7^ nor the International Classification of Diseases (ICD-10)^8,9^ trichotillomania and excoriation disorder have been explicitly mentioned in both classifications as separate conditions. DSM-5, in addition, uses the category of “other specified” for other BFRB and lists here nail biting and lip biting. Categorization of both trichotillomania and excoriation disorder changed from the category of impulse control disorder (ICD) in DSM-IV-TR to conditions under the heading of „obsessive-compulsive and related disorders” in DSM (DSM-5). However, there is probably a combination of both impulsive and compulsive components^10^ with a predominance of the compulsive element^11^. This is also reflected in a tentative division of BFRB into focused and automatic behaviors^12,13^. Focused BFRB are considered deliberate actions that are usually performed in response to negative emotions such as stress and anxiety and correlate with emotion regulation problems^14^. As a result, the motive behind its performance is to release these negative emotions^13,15^. In contrast, automatic BFRB are thought to be performed without full awareness and are usually pleasurable. They are mainly performed during relaxation or when an individual is bored (i.e. while reading or watching TV). Often, individuals do not realize that they execute such behaviors unless they are pointed out or after their performance. Overall, focused BFRB have more negative impact on quality of life than automatic BFRB with increased risk for depression^16,17^. However, recent studies indicate that many patients experience both types of BFRB with a fluctuating course depending on the situation.

### Differential diagnoses of BFRB: SIB and stereotypies

Main differential diagnoses of BFRB are motor stereotypies and SIB^18^. Frequently, however, it remains challenging due to the co-existence of several of these phenomena in one single person and given the fact that especially for BFRB and SIB there are no clear and generally accepted definition. Similar to BFRB, the status of SIB is inconclusive: while in DSM-5 it is classified under the category of “personal risk factors, not elsewhere classified”^7^ in contrast, in ICD-10 SIB is categorized under the spectrum of “symptoms and signs involving emotional state”. Stereotypies, on the other hand, are defined as repetitive movements and/or vocalizations with typical examples of body rocking or hand flapping^19^. As they typically start in early childhood and co-exist with other neurodevelopmental conditions such as autism spectrum disorder, according to DSM-5 stereotypes belong to the spectrum of neurodevelopmental conditions. This stays in line with the categorization in ICD-10 where it is classified under the umbrella of “other behavioral and emotional disorders with onset usually occurring in childhood and adolescence”.

Although belonging to different categories in DSM-5 and ICD-10, the frontiers between BFRB, SIB, and stereotypies is widely discussed. Accordingly, it has been speculated that for the upcoming classification of DSM-6, these three categories might be grouped together. Particularly controversially discussed and clinically difficult is the differentiation between BFRB and SIB^15,18,20^. The main feature helping to distinguish between these two phenomena is the intention behind the execution of the behavior as in the case of BFRB it is mainly performed to relieve tension or stress, while in the case of SIB, there is typically an intention of self-injury or harm. However, individuals with SIB do not always endorse such feelings, especially those with only mild SIB^21^ and patients with TS^22^. In addition, there are significant culture-dependent differences in reports of such intentions^23^. A preceding urge can occur in both BFRB and SIB, especially in non-suicidal SIB (NSSI)^24^ and therefore is not a useful distinguishing feature.

### Comorbid BFRB in tic disorders

There is a complex relationship between tics and BFRB that is heavily under investigated^25–29^. This association is even more complex given that in some patients with TS, not only BFRB co-exists, but also OCD, ADHD, and SIB. Studies on BFRB in patients with TS focussed mainly on specific subtypes including trichotillomania, excoriation disorder, and nail biting^26–29^. Lamothe et al.^26^ conducted a systematic review to investigate the relationship between BFRB and both tics and OCD and found that trichotillomania is more related to tics than OCD. This is in line with findings by Rozeman et al.^28^ who compared pediatric patients with tics (*n* = 29), trichotillomania (*n* = 30), and OCD (*n* = 30) and also found that trichotillomania is more similar to tics than OCD. Greenberg et al.^27^ investigated the prevalence of trichotillomania and excoriation disorder in patients with tics (*n* = 811) and found a prevalence rate of only 3.8% for trichotillomania, but of 13.0% for excoriation disorder. In a univariate analysis, female sex, OCD, and severity of both tics and OCD were associated with BFRB. In multivariable analyses, only lifetime worst-ever motor tic severity remained significantly associated with trichotillomania. Female sex, co-occurring OCD, ADHD, and motor tic severity remained independently associated with excoriation disorder. In a recent large study from Taiwan^29^ prevalence and correlates of nail-biting was investigated in patients with tics (*n* = 535 with TS and *n* = 230 with PTD) compared to controls (*n* = 1460 people without neurological or psychiatric disorders). Nail biting was more commonly observed in patients with TS (56.6%) than in patients with PTD (27.4%) and controls (15.0%). Nail biting was also more common in patients with TS with comorbid ADHD than in those without, but the starting age was significantly later in those with concomitant ADHD than those without. In patients with TS, the onset of nail biting was earlier than that of tics, regardless of ADHD status.

In our study, we investigated the clinical profile of BFRB in adult patients with chronic tic disorders as well as its association with tic severity and psychiatric comorbidities. In addition, we compared patients with comorbid SIB and comorbid BFRB. We hypothesized that BFRB is overall a frequent phenomenon in patients with tics and correlates with tic severity and OCD. Compared to SIB, we hypothesized that BFRB is more related to the OCD spectrum, while SIB is more associated with tics.

## Results

Of 123 patients included, current BFRB behaviors and urges were reported by *n* = 59 (48%).

*Currently present BFRB behaviors* (59/123, 48%) had the following distribution: *n* = 12/123 (9.7%) reported trichotillomania, *n* = 26/123 (21.1%) skin picking, *n* = 19/123 (15.44%) bruxism and *n* = 20/123 (16.3%) nail (More than one answer may apply. See Table 1 for further clarification).

**Table 1:** Summary statistics for current type of body focused repetitive behaviors.

Type of BFRB	Subtype	Frequency* (*N*/%)
Trichotillomania (12/9.7%)	Pulling out hair	7/5.7%
	Pulling out eyelashes and eyebrows	3/2.4%
	Pulling out hair from other parts of the body	8/6.5%
Skin picking (26/21.1%)	Scratching the skin	13/10.6%
	Scratching the wounds	28/22.8%
Bruxism (19/15.44%)	Strong biting of objects with destruction of teeth	1/0.8%
	Clenching the teeth to destroy it	10/8.1%
	Strong heating teeth with each other	14/11.4%
	Clenching teeth with hands	1/0.8%
	Hitting teeth with objects	1/0.8%
Nail biting (20/16.3%)	Nail Biting	20/16.3%
	Toe biting	2/1.6%

* More than one answer may apply.

*Current urge* to perform any BFRB (*n* = 59/123, 48%) had the following distribution: *n* = 13/123 (10.6%) trichotillomania, *n* = 33/123 (26.8%) skin picking, *n* = 27/123 (22%) bruxism, and *n* = 23/123 (18.7%) nail (More than one answer may apply. See Table 2 for further clarification).

**Table 2:** Summary statistics for current type of urge to perform body focused repetitive behaviors (BFRB), *N* = 123.

Type of BFRB	Subtype	Frequency* (*N*/%)
Trichotillomania (13/10.6%)	Pulling out hair	8/6.5%
	Pulling out eyelashes and eyebrows	3/2.4%
	Pulling out hair from other parts of the body	8/6.5%
Skin picking (33/26.8%)	Scratching the skin	11/8.9%
	Scratching the wounds	30/24.4%
Bruxism (27/22.0%)	Strong biting of objects with destruction of teeth	6/4.9%
	Clenching the teeth to destroy it	16/13.0%
	Strong heating teeth with each other	16/13.0%
	Clenching teeth with hands	1/0.8%
	Hitting teeth with objects	1/0.8%
Nail biting (23/18.7%)	Nail Biting	22/17.9%
	Toe biting	6/4.9%

*More than one answer may apply.

Only 12/123 (20%) individuals experienced an *urge* without subsequent behaviors compatible with BFRB. The vast majority (80%) had both *BFRB urges* and *behaviors.* Only 3/123(5.1%) individuals experienced current *behaviors* without precipitating *urges*.

Additional results regarding the historical presence of urges and/or behaviors compatible with BFRB are mentioned in the Supplementary Material 1 and Supplementary Tables 1 and 2.

When comparing patients reporting currently present BFRB behaviors and those without, patients with BFRB behaviors had higher scores in the following scales: OCI-R (*p* = 0.015), BSL-23 (*p* = 0.091), and GTS-QOL (*p* = 0.0185) (for further details, please consult Table 3 and Supplementary Table 3). Individuals with both current BFRB urges *and* behaviors compared to those without were characterized by: higher prevalence of female sex (*p* = 0.0042), more frequent prevalence of OCD (according to patients’ reports) (*p* = 0.0258), higher tic severity indicated by the following results: total number of tics in ATQ (*p* = 0.0313), number of vocal tics (*p* = 0.0219), number of complex tics (*p* = 0.0380), number of complex motor tics (*p* = 0.0397), higher severity of OCS as measured with OCI-R (*p* = 0.0027), higher results in BSL-23 (*p* = 0.0189) as well as lower quality of life according to GTS-QOL (*p* = 0.0133) (for further details, please consult Table 4 and Supplementary Table 4).

**Table 3:** Comparison between patients with (*N* = 59) and without (*N* = 64) *current* body focused repetitive behaviors (BFRB) without BFRB urge. Only statistically significant results are provided while an overview of all results is provided in supplementary table 4.

Variable	With BFRB behavior	No behavior	*P* value
OCI (mean)	38.63 SE 1.82, 95% CI 34.97–42.30	31.54 SE 1.30, 95% CI 28.95-34.133	0.0015
BSL-23 (mean)	20.67 SE 2.82, 95% CI 15.00-26.35	12.99 SE 1.44, 95% CI 10.11–15.87	0.0091
GTS-QOL (mean)	40.04 SE 3.08, 95% CI 33.86–46.22	30.88 SE 2.37, 95% CI 26.16–35.60	0.0185

SE – standard error, CI – confidence interval, OCI – the Obsessive-Compulsive Inventory, BSL-23 – the Borderline Symptom List, GTS QOL – the Gilles de la Tourette Quality of Life Scale; all scales were self-assessments.

**Table 4:** Comparison between patients with (*N* = 59) and without (*N* = 64) *current urges* to perform body focused repetitive behaviors (BFRB), but without BFRB *behavior*. Only statistically significant results are provided while an overview of all results is provided in supplementary table 3.

Variable	With BFRB urge	No urge	*P* value
Sex (n,%)	32/82, 39% (male) 27/41, 65.85% (female)	50/82, 60.98% (male) 14/41, 34.15% (female)	0.0047
ATQ (mean) number of tics total	13.19 SE 0.75, 95% CI 11.69–14.68	10.94 SE 0.69, 95% CI 9.55–12.33	0.03
Vocal tics	4.81 SE 0.42, 95% CI 3.97–5.66	3.48 SE 0.37, 95% CI 2.74–4.23	0.19
Complex tics	3.59 SE 0.44, 95% CI 2.71–4.48	2.47 SE 0.34, 95% CI 1.78–3.15	0.44
Complex motor tics	1.15 SE 0.136, 95% CI 0.88–1.43	0.8 SE 0.11, 95% CI 0.57–1.02	0.45
Vocal tics	7.16 SE 0.381, 95% CI 6.40–7.93	6.77 SE 0.299, 95% CI 6.17–7.37	0.037
ATQ (mean) frequency total	31.15 SE 17.67, 95% CI 26.55–35.76	25.08 SE 15.67, 95% CI 21.16–28.99	0.46
Vocal tics	8.66 SE 0.92, 95% CI 6.82–10.50	6.19 SE 0.78, 95% CI 4.63–7.75	0.0415
Simple vocal tics	6 SE 0.61, 95% CI 4.78–7.22	4.31 SE 0.52, 95% CI 3.28–5.35	0.04
Complex tics	2.90 SE 0.43, 95% CI 2.15–3.75	1.92 SE 0.307, 95% CI 1.308–2.536	0.0424
Complex motor tics	2.95 SE 0.401, 95% CI 2.15–3.75	1.92 SE 0.307, 95% CI 1.308–2.536	0.0424
BAI (mean)	15.27 SE 1.37, 95% CI 12.52–18.02	10.58 SE 1.28, 95% CI 8.01–13.14	0.0138
ADHS-SB (mean)	2.24 SE 0.21, 95% CI 1.81–2.66	1.65625 SE 0.18, 95% CI 1.29–2.02	0.0394
BDI (mean)	15.69 SE 1.37, 95% CI 12.96–18.43	11.89 SE 1.16, 95% CI 9.58–14.20	0.0349
OCI (mean)	37.68 SE 1.65, 95% CI 34.38–40.98	31.31 SE 1.40, 95% CI 28.52–34.11	0.0037
BSL-23 (mean)	20.24 SE 2.52, 95% CI 15.20-25.27	12.19 SE 1.42, 95% CI 9.36–15.02	0.0052
GTS-QOL (mean)	41.41 SE 2.84, 95% CI 35.72–47.09	28.19 SE 2.34, 95% CI 23.52–32.86	0.0004
GTS VAS (mean)	51.36 SE 2.76, 95% CI 45.83–56.88	62.34 SE 2.53, 95% CI 57.29–67.39	0.0039

SE – standard error, CI – confidence interval, ADD - attention deficit disorder, ATQ – the Adult Tic Questionnaire, BAI – the Beck Anxiety Inventory, ADHS-SB - ADHS-Selbstbeurteilungsskala, BDI – the Beck Depression Inventory, OCI – the Obsessive-Compulsive Inventory, BSL-23 – the Borderline Symptom List, GTS QOL – the Gilles de la Tourette Quality of Life Scale, GTS VAS – the Visual Analogue Scale for Quality of Life; all scales were self-assessments.

When comparing participants with currently present BFRB urge (*N* = 59) and those without (*N* = 64), the following differences emerged: urge for BFRB was more frequently reported by females (*p* = 0.0047), patients with urge have more common comorbid OCD (*p* = 0.0058), higher severity of tics as measured with ATQ Total Scale (*p* = 0.03), higher frequency of vocal tics (*p* = 0.019), higher number of complex tics (*p* = 0.044) and in particular complex motor tics (*p* = 0.046), greater tic frequency (*p* = 0.046), in particular frequency of vocal tics (*p* = 0.0415), intensity of complex tics (*p* = 0.0424), and intensity of complex motor tics (*p* = 0.0424). In addition, patients who reported an urge to perform BFRB had higher (more severe) scores in the following scales assessing psychiatric comorbidities: anxiety as assessed by BAI (*p* = 0.0138), ADHD as assessed by ADHS-SB (*p* = 0.0394), depression as assessed by BDI (*p* = 0.0394), obsessive-compulsive symptoms (OCS) as assessed by OCI-R (*p* = 0.0037), and features typical for borderline personality disorder as assessed by BSL-23 (0.0052). Finally, patients who reported having an urge to perform BFRB had lower quality of life as measured with GTS-QOL (*p* = 0.0004) and GTS-VAS (0.0039) (for further details, please consult Table 5 and Supplementary Table 5).

**Table 5:** Comparison between patients with both current urge and behavior related to body focused repetitive behaviors (BFRB) and those with neither BFRF urge nor BFRB behavior. Only statistically significant results are provided while an overview of all results is provided in supplementary table 5.

Variable	Both BFRB urge and behavior	Neither urge nor behavior	*P* value
Sex (n,%)	38/82, 46.3% (male) 30/41, 73.2% (female)	44/82, 53.7% (male) 11/41, 26.8% (female)	0.0042
OCD (n,%)	23/59, 39%	9/64, 14.1%	0.0258
ATQ number of tics total (mean)	13.01 SE 0.69, 95% CI 11.63–14.39	10.78 SE 0.75, 95% CI 9.27–12.29	0.313
Vocal tics	4.72 SE 0.38, 95% CI 3.95–5.46	3.4 SE 0.41, 95% CI 2.57–4.23	0.219
Complex tics	3.53 SE 0.40, 95% CI 2.72–4.33	2.36 SE 0.36, 95% CI 1.63–3.09	0.380
Complex motor tics	1.13 SE 0.13, 95% CI 0.88–1.38	0.76 SE 0.12, 95% CI 0.52–1.007	0.397
Simple vocal tics	2.31 SE 0.17, 95% CI 1.97–2.65	1.8 SE 0.21 95% CI 1.38- 2.22	0.0596
OCI (mean)	37.33 SE 1.49, 95% CI 34.36–40.29	30.71 SE 1.54, 95% CI 27.63–33.79	0.0027
BSL-23 (mean)	19.10 SE 2.24, 95% CI 14.64–23.56	12.27 SE 1.60, 95% CI 9.07–15.47	0.0189
GTS-QOL (mean)	38.76 SE 2.62, 95% CI 33.54–43.99	29.29 SE 2.66, 95% CI 23.95–34.63	0.0133

SE – standard error, CI – confidence interval, ATQ – the Adult Tic Questionnaire, OCI – the Obsessive-Compulsive Inventory, BSL-23 – the Borderline Symptom List, GTS QOL – the Gilles de la Tourette Quality of Life Scale, OCD - obsessive compulsive disorder.

Finally, we compared patients with current BFRB behaviors (*n* = 12) and those with SIB behaviors other than BFRB (*n* = 34). Patients with SIB were significantly younger (*p* = 0.0196), suffered more frequently from ADHD (*p* < 0.001), and had overall higher total tic severity (*p* = 0.0020) including higher number of tics (*p* = 0.0013) and different subtypes of tics: motor tics (*p* = 0.0013), vocal tics (*p* = 0.0109), complex tics (*p* = 0.0008), complex motor tics (*p* = 0.0089), complex vocal tics (*p* = 0.0007), simple tics (*p* = 0.0271), and simple motor tics (*p* = 0.0035); higher total tic frequency (*p* = 0.0054) and frequencies of motor tics (*p* = 0.0013), complex tics (*p* = 0.0051), complex motor tics (*p* = 0.0156), complex vocal tics (*p* = 0.0047), simple tics (*p* = 0.0176), and simple motor tics (*p* = 0.0018); and higher total tic intensity (*p* = 0.0027) and intensities of motor tics (*p* = 0.0018), vocal tics (*p* = 0.0203 ), complex tics (*p* = 0.0047), complex motor tics (*p* = 0.0047), complex vocal tics (*p* = 0.0010), simple tics (*p* = 0.0477), and simple motor tics (*p* = 0.0055) (for further details, please consult Table 6 and Supplementary Table 6).

**Table 6:** Comparison between patients with current body focused repetitive behaviors (BFRB behaviors) and non-BFRB self-injurious behaviors (SIB behaviors) (*n* = 71). *N* = 37 have both BFRB and non-BFRB related SIB, *N* = 12 pure BFRB, and *N* = 34 pure non-BFRB related SIB. Only statistically significant results are provided while an overview of all results is provided in supplementary table 6.

Variable	Non BFRB related SIB	BFRB	*P* value
Age (mean)	31.91 SE 2.39, 95% CI 27.06–36.77	44.00 SE 5.02, 95% CI 32.94–55.06	0.0196
ADHD (n,%)	6/34, 17.65%	0/12, 0%	0.000
ATQ number of tics total (mean)	14.35 SE 0.94, 95% CI 12.45–16.26	8.33 SE 1.25, 95% CI 5.58–11.08	0.13
Motor tics	8.82, SE 0.47, 95% CI 7.93–9.84	5.58, SE 0.93, 95% CI 3.53–7.64	0.13
Vocal tics	5.47 SE 0.58, 95% CI 4.28–6.66	2.75, SE 0.45, 95% CI 1.77–3.73	0.0109
Complex tics	4.59, SE 0.55, 95% CI 3.47–5.71	1.08, SE 0.50, 95% CI − 0.016 2.18	0.0008
Complex motor tics	1.41 SE 0.18, 95% CI 1.04–1.79	0.5 SE 0.19, 95% CI 0.07–0.93	0.0089
Complex vocal tics	3.18 SE 0.41, 95% CI 2.35-4.00	0.58 SE 0.34, 95% CI -0.16- 1.32	0.07
Simple tics	9.76 SE 0.56, 95% CI 8.62–10.91	7.25 SE 0.95, 95% CI 5.17–9.33	0.0271
Simple motor tics	7.47 SE 0.37, 95% CI 6.73–8.22	5.08 SE 0.79, 95% CI 3.34–6.83	0.0035
ATQ frequency total (mean)	33.74 SE 2.95, 95% CI 27.74–39.73	18 SE 3.43, 95% CI 10.46–25.54	0.0054
Motor tics	24.32 SE 1.97, 95% CI 20.31–28.34	11.92 SE 2.29, 95% CI 6.88–16.95	0.0013
Complex tics	6.97 SE 1.10, 95% CI 4.74–9.20	1.42 SE 0.54, 95% CI 0.22–2.61	0.0051
Complex motor tics	3.09 SE 0.47, 95% CI 2.13–4.05	1 SE 0.37, 95% CI 0.19–1.81	0.0156
Complex vocal tics	3.88 SE 0.68, 95% CI 2.50–5.27	0.42 SE 0.23, 95% CI 0.09–0.92	0.0047
Simple tics	26.76 SE 2.20, 95% CI 22.3-31.23	16.58 SE 3.08, 95% CI 9.80-23.36	0.0176
Simple motor tics	21.24 SE 1.69, 95% CI 17.79–24.68	10.92 SE 2.03, 95% CI 6.44–15.39	0.0018
ATQ intensity total (mean)	35.5 SE 3.02, 95% CI 29.35–41.65	18.08, SE 3.354008, 95% CI 10.70-25.47	0.0027
Motor tics	21.85 SE 1.61, 95% CI 18.58–25.13	11.67, SE 2.36, 95% CI 6.47–16.87	0.0018
Vocal tics	13.65 SE 1.71, 95% CI 10.18–17.12	6.42 SE 1.39, 95% CI 3.36–9.48	0.203
Complex tics	3.44 SE 0.54, 95% CI 2.34–4.54	0.67, SE 0.28, 95% CI 0.04–1.29	0.0047
Complex motor tics	3.44 SE 0.54, 95% CI 2.34–4.54	0.67, SE 0.28, 95% CI 0.04–1.29	0.47
Complex vocal tics	8.82 SE 1.28, 95% CI 6.21–11.44	1.08, SE 0.61, 95% CI 0.26–2.42	0.0010
Simple tics	23.24 SE 1.71, 95% CI 19.76–26.71	16.33, SE 3.02, 95% CI 9.68–22.99	0.0477
Simple motor tics	18.41 SE 1.30, 95% CI 15.78–21.05	11, SE 2.18, 95% CI 6.19–15.81	0.0055
ATQ Total (mean)	83.59 SE 6.53, 95% CI 70.31–96.87	44.42, SE 7.70, 95% CI 27.46–61.37	0.0020

SE – standard error, CI – confidence interval, ATQ – the Adult Tic Questionnaire, ADHD - attention deficit hyperactivity disorder, BFRB - body focused repetitive behaviors, SIB - self-injurious behaviours; all scales wereself-assessments.

## Discussion

Our study is the first to investigate BFRF and in addition to compare SIB and BFRB in patients with chronic tic disorders. We also provide overall characteristics of the phenomenology and clinical correlates of BFRB in this group of patients. We found that BFRB (urge and behavior) is a common comorbidity in patients with chronic tic disorders occurring in nearly 50%, especially in females, individuals with comorbid obsessive-compulsive symptoms (OCS) or OCD, and higher tic severity. Both urges and behaviors to perform BFRB had a negative impact on quality of life. From our data it is suggested that SIB and BFRB belong to the same spectrum with SIB indicating a more severe clinical manifestation, since SIB was found in younger patients with more severe tics and comorbid ADHD.

The prevalence rate of BFRB in our sample was high with half of patients and in line with some studies (e.g. study from Canada with prevalence of 53%)^30^, but much higher compared to other reports (between 2.4-56.6%)^26,27,29,30^. This difference could be related to several factors. Firstly, different definitions of BFRB may have been used such as predefined symptom severity, as in some studies, only BFRB causing significant impairment of quality of life were assessed. For example, in a study from Canada^30^ 53% of patients reported having any type of BFRB, but only 12% fulfilled diagnostic criteria for BFRB according to DSM-5. In addition, some studies only focused on particular BFRB such as nail-biting^29^ trichotillomania^26^ or excoriation disorder^27^. Secondly, study populations may vary, as our study included only adults. Thirdly, geographical and cultural discrepancies could also have an impact as it is known that symptom disclosure diverges between cultures^31,32^. In addition, there are differences in tic phenomenology across cultures with, for example, more frequent prevalence of coprolalia in individuals from Latin America and lowest in Asia which could be related to social acceptance of symptoms^33^. Fourthly, different settings and recruitment strategies may influence the results: while the majority of studies on BFRB had been conducted in specialized tic disorders clinics, which may have caused a selection bias, our study was conducted online which, in spite of its limitations, could be more representative of the real-life situation. Fifthly, preliminary data indicate that there are differences between TS, CMT, and PTD in terms of profile and severity of psychiatric comorbidities with TS being the most severe on the spectrum^3^ suggesting that, depending on the type of tic disorders, prevalence of BFRB can also vary. Possible differences in BFRB prevalence among patients with different types of tic disorders have been also suggested in a recent study from Taiwan in which nail biting was more commonly observed in patients with TS (56.6%) than in those with PTD (27.4%)^29^. This hypothesis is in line with our results demonstrating an association between tic severity and BFRB.

In line with recent studies in patients with tics^30^but also in patients without tics^5,34^ in our sample BFRB was more frequent in females than in males, especially skin picking and trichotillomania^5,35^. Furthermore, it is well known that psychiatric comorbidities have a significant impact on quality of life in patients with tic disorders^36^. Accordingly, we were able to demonstrate that also BFRB has a negative effect on patients’ quality of life, similar to comorbid SIB in patients with tics^36^ but also to impairment of quality of life in patients with BFRB without tics^37^.

In terms of clinical correlations, we found that BFRB were overall correlated with OCS/OCD and higher tic severity. Similarly, Bhikram et al.^30^ have demonstrated a correlation between tic severity and OCD, respectively, and the prevalence of skin picking disorder and hair pulling disorder in patients with tics. Hsueh et al.^29^ found a higher prevalence of nail biting in patients with tics compared to healthy controls and nail biting was associated with comorbid ADHD. However, different types of BFRB have been found to be associated with different psychiatric comorbidities. For example, trichotillomania and skin picking are mainly associated with OCD spectrum, while nail-associated BFRB such as habit-tic nail deformity, onychophagia, and onychotillomania have been mainly associated with anxiety^38,39^. So far, it is unknown whether BFRB is closer related to tics or OCD in patients with tic disorders. In a recent review by Lamothe et al.^26^ the authors concluded that trichotillomania is more related to TS than OCD.

Since SIB is a common symptom in patients with borderline personality disorder, in our original SIB study^40^we also collected data regarding the presence of this disorder (using the BSL-23) to avoid false positive results. With respect to BFRB, we found that patients with an urge and/or behavior to perform BFRB had more pronounced features compatible with borderline personality disorder in comparison to patients with tics without BFRB. However, based on our data we can exclude that both SIB and BFRB in patients with tic disorders is only caused by co-existing borderline personality disorder. Our findings stay in line with a recently published study by Grant et al.^41^ demonstrating that 37.4% of adult patients with BFRB screened positive for probable diagnosis of borderline personality disorder. In addition, patients who screened positive for possible borderline personality disorder reported - among several other symptoms - worse pulling and picking symptoms. There is preliminary evidence suggesting that there are differences between different types of BFRB and personality traits with excoriation disorder, but not trichotillomania, being more associated with borderline personality traits^42^.

Finally, we investigated differences between SIB and BFRB and found that SIB is more prevalent in younger individuals with more severe tics and comorbid ADHD suggesting that BFRB may belong to the same spectrum with SIB being the more severe clinical manifestation. However, differentiation between BFRB and SIB remains challenging, since there are no clear definitions and differential criteria available. In a recent large study, Mathew et al.^20^ compared individuals with NSSI (*n* = 165) and different types of BFRB (hair pulling (*n* = 102), skin picking (*n* = 216), nail picking (*n* = 253), nail biting (*n* = 487), and cheek biting (*n* = 300)). Individuals with NSSI reported more frequently engaging in the behavior for social-affective reasons (i.e., to get out of doing something or receive attention from others) and that they performed these behaviors to regulate tension and feelings of emptiness, while SIB execution provided significant symptom relief. Contrary to that, BFRB was mainly performed automatically without self-reflection and awareness, mainly to alleviate boredom. Some individuals also reported that BFRB were performed to improve physical appearance. In the same study^20^ it has been found that SIB is associated with higher results on scales measuring stress, anxiety, depression, and harm avoidance confirming our hypothesis that SIB is overall associated with more severe symptomatic phenotype. Moritz et al.^15^ investigated intentions for the performance of BFRB in order to identify criteria that help to distinguish BFRB from SIB and motor stereotypies. They found that the main reason to perform BFRB is to improve stress, boredom, and pleasure. However, even one third of participants were not able to describe an exact motivation behind the execution of BFRB, which complicates differential diagnosis, especially with SIB. This is the more a problem given the fact that in some cases both phenomena co-occur. However, while some individuals have negative associations with their BFRB, it is not always the case as sometimes they are neutral to the person performing them. In our study, we did not investigate intentions behind execution of each type of behaviors, but only used the kind of symptom to differentiate between SIB and BFRB. From our results, it is suggested that SIB and BFRB in patients with tics should be treated within the same spectrum rather than constitute two different phenomena.

Our study has some limitations. Firstly, data used in this study had been collected online and, therefore, diagnoses could not be confirmed by a clinician. Therefore, it cannot entirely excluded that patients with other disorders (including functional tic-like behaviors) participated in the survey. Secondly, due to the online design, only self-administered scales could be used. For example, the ATQ and the OCI-R, respectively, have been used for the assessment of tics and OCD, instead of the gold standard for tic assessment, the Yale Global Tic Severity Scale (YGTSS), and for OCD, the Yale-Brown Obsessive Compulsive Inventory (Y-BOCS). Thirdly, the study was originally designed to measure SIB in patients with chronic tic disorders and validate a new instrument for SIB assessment, the SIBS-T.Only retrospectively, we differentiated between SIB and BFRB (urge and behavior) based on clinical symptomatology. Fourthly, in this study, only adults had been included and therefore it is unclear whether results can be transferred to children populations. Sixthly, since people with BFRB may feel ashamed, answers my not be truthful. However, in general people provide more truthful answers in online surveys compared to personal interviews^43^.

## Methods

For this study we used data collected for another project dedicated to SIB and the development and validation of a new rating scale for the assessment of SIB in patients with tic disorders, the Self-injurious Behavior Scale for Tic Disorders (SIBS-T)^40^. For the purpose of that study, the following definition for SIB had been used^7,44^: “SIB are defined as an urge to or a behavior of injuring oneself against one’s own will. SIB are not performed intentionally and are not the result of an accident. They can lead to injury or harming of one’s own body without having an intension of self-harm or suicide (suicidality). These behaviors must be performed even though their senselessness and self-damage are recognized”^44–49^. This definition has been based on extensive literature review that was done by the authors also in the previous publications^40,44^ and updated currently, clinical experience, and experts’ opinion and stays in line with DSM-5 classification.

This former project was a prospective, cross-sectional study conducted online via SoSciSurvey. We collected anonymously demographic data (sex, age, level of education), asked for current medication and other treatments, and collected information about previously diagnosed psychiatric disorders, i.e., ADHD, OCD/OCS, depression, anxiety, sleep disorder, eating disorder, alcohol or drug addiction, and personality disorder, especially borderline personality disorder. Borderline personality disorder - although not a typical comorbidity in patients with tic disorders - was included, since SIB is a common phenomenon in this group of patients in order to avoid false positive results. If any psychiatric comorbidity had been diagnosed, participants were asked whether symptoms are still present and clinically relevant. In order to assess tic severity and severity of psychiatric comorbidities, a number of scales had been implemented. Since the study was performed online, only self-assessments could be used: (i) ATQ for assessment of tics^50^ (ii) Self-Assessment Scale of ADHD (ADHS-Selbstbeurteilungsbogen, ADHS-SB) to measure severity of ADHD symptoms^51^ (iii) Obsessive–Compulsive Inventory-Revised (OCI-R)^52^ for assessment of OCD, (iv) Scale of Impulsive Behaviors (Skala Impulsives-Verhalten-8, I-8)^53^ for evaluation of impulsiveness, (v) Beck Depression Inventory (BDI-II)^54^ for evaluation of depression, (vi) Beck Anxiety Inventory (BAI)^55^ for evaluation of anxiety, (vii) short version of the Borderline Symptom List (BSL-23)^56^ for measurement of features typical for borderline personality disorder, and (viii) Rage Attack Questionnaire-Revised (RAQ-R)^57^ for evaluation of rage attacks. To assess quality of life, Gilles de la Tourette Syndrome Quality of Life Scale (GTS-QoL)^58^ and Visual Analogue Scale (QoL-VAS)^59^ were used.

Overall, 123 adult patients with tic disorders were included, 82 of whom (66.7%) were males, the mean age of participants was 36.5 (SD = 14.18) years. In terms of clinical diagnosis, (*n* = 102, 82.9%) had pre-diagnosed TS, *n* = 13 (10.6%) chronic tic disorder (CTD), and *n* = 8 (6.8%) unspecified tic disorder. While this study was based on patients’ self-report, recruitment was mainly based on our Tourette Outpatient Clinic and nearly half of the patients (*n* = 55, 44.7%) indicated that the diagnosis had been made in our clinic. More than half of patients (56.1%, *n* = 69) were taking medications for tics and/or psychiatric comorbidities, most frequently aripiprazole (*n* = 20, 29%) and cannabis-based medicines (*n* = 17, 24.6%). The profile of comorbidities included depression (*n* = 33, 26.8%), OCD (*n* = 32, 26%), anxiety (*n* = 23, 18.7%), ADHD (*n* = 15, 12.2%), sleeping problems (*n* = 14, 11.4%), and autism spectrum disorder (*n* = 7, 5.7%). Further demographic characteristics of this cohort as well as details regarding clinical characteristics of SIB can be consulted in the original study by Szejko et al.^40^.

The SIBS-T includes a checklist with 32 different SIB symptoms clustered in seven categories (hitting/pushing, scratching/pinching, eye-related SIB, biting/licking, tooth-related SIB, trichotillomania, and skin burning) assessing five aspects of SIB: number, frequency, intensity, control over SIB, and impairment, each rated on a scale ranging from 0 to 4. For each SIB both preceding urges and behavior were considered as well as currently and historical presence of symptoms. Further details regarding inclusion criteria, recruitment and sample calculation can be consulted in the original paper^40^.

For the current study, based on the clinical symptomatology and a systematic literature review we selected 12 symptoms out of the 32 former “SIB symptoms”^40^ separated them from SIB, and classified them as BFRB rather than SIB. This selection was based on DSM-5 classification. A systematic review of the literature performed by the authors as part of another independent project of a scoping review of BFRB in patients with tics (Rylska et al., data unpublished)^60–62^. The protocol of this study can be consulted on the Open Science Framework (https://osf.io/wt9x5/)^61^. Briefly, following the search in Pubmed and Scopus databases, we identified 22,307 abstracts and after blinded screening by three reviewers and conflict resolution, 11 were included in the final analysis^26,27,29,30^,^63–68^. After full-article screening, the following symptoms were classified as BFRB: pulling out hair, pulling out eyelashes and eyebrows, pulling out hair from other parts of the body, scratching the skin, scratching wounds, destruction of teeth due to biting of objects, teeth clenching, vigorous clenching or grinding of the teeth, holding the jaw tightly with both hands while gritting the teeth, hitting teeth with objects, nail biting, and toe biting.

Summary statistics were used to analyse distribution of current and historical presence of urge and/or behavior compatible with BFRB. T-test or logistic regression were used to compare demographic features, distribution of psychiatric comorbidities, as well as severity of tics and psychiatric comorbidities between: (i) patients with and without current urge to perform BFRB; (ii) patients with and without current BFRB; (iii) patients with either urge or behavior compatible with BFRB; and (iv) patients with either BFRB or SIB. For the last comparison, we compared patients with pure BFRB (without co-existing SIB) and pure SIB (without co-existing BFRB).

## Electronic supplementary material

Below is the link to the electronic supplementary material.

Supplementary Material 1

Supplementary Material 2

Supplementary Material 3

Supplementary Material 4

Supplementary Material 5

Supplementary Material 6

Supplementary Material 7

## Data Availability

Data supporting this manuscript could be received on request from the corresponding author.
