# Supplementary material for: Frequency of body focused repetitive behaviors and comparison to self-injurious behaviors in patients with tic disorders
Source: Sci Rep. 2025 Aug 25;15:31238. doi: 10.1038/s41598-025-12023-5 (PMC12379270; doi:10.1038/s41598-025-12023-5)
Supplement: Supplementary file 1 — Supplementary Material 1 [file 41598_2025_12023_MOESM1_ESM.docx]

Supplementary Table 1. Summary statistics for past body focused repetitive behaviors.

| **Type of BFRB** | **Subtype** | **Frequency**  (N/%) |
| --- | --- | --- |
| **Trichotillomania**  (n=12, 9.7%) | Pulling out hair | 6/4.9% |
|  | Pulling out eyelashes and eyebrows | 5/4.1% |
|  | Pulling out hair from other parts of the body | 6/4.8% |
| **Skin picking**  (n=45, 36.6%) | Scratching the skin | 13/10.6% |
|  | Scratching the wounds | 17/13.8% |
| **Bruxism**  (n=39, 31.7%) | Strong biting of objects with destruction of teeth | 13/10.6% |
|  | Clenching the teeth to destroy it | 17/13.8% |
|  | Strong heating teeth with each other | 21/17.1% |
|  | Clenching teeth with hands | 7/5.7% |
|  | Hitting teeth with objects | 1/0.8% |
| **Nail biting**  (n=19, 15.44%) | Nail Biting | 16/13% |
|  | Toe biting | 9/7.3% |

More than one answer may apply.
