# Supplementary material for: Frequency of body focused repetitive behaviors and comparison to self-injurious behaviors in patients with tic disorders
Source: Sci Rep. 2025 Aug 25;15:31238. doi: 10.1038/s41598-025-12023-5 (PMC12379270; doi:10.1038/s41598-025-12023-5)
Supplement: Supplementary file 3 — Supplementary Material 3 [file 41598_2025_12023_MOESM3_ESM.docx]

Supplementary Table 3. Comparison between patients with (N=59) and without (N=64) *current* body focused repetitive behaviors (BFRB) without BFRB urge.

| Variable | With BFRB behavior | No behavior | P value |
| --- | --- | --- | --- |
| Age (mean) | 36.27 SE 2.066, 95% CI 32.11-40.42 | 36.70 SE 1.64, 95% CI 33.44-39.96 | 0.8678 |
| Sex (n,%) | 31/82, 37.8% (male)  18/41, 43.9% (female) | 51/82, % (male)  23/41, % (female) | 0.5159 |
| ADD (n,%) | 2/59, 3.4% | 5/64, 7.8% | 0.4974 |
| ADHD (n,%) | 7/59, 11.9% | 8/64, 12.5% | 0.5669 |
| OCD (n,%) | 15/59, 25.4% | 17/64, 26.6% | 0.3469 |
| Depression (n, %) | 12/59, 20.3% | 21/64, 32.8% | 0.6325 |
| Anxiety (n, %) | 8/59, 13.6 % | 15/64, 23.4% | 0.5805 |
| Sleeping problems (n, %) | 5/59, 8.5% | 9/64, 14.1% | 0.7364 |
| ATQ number of tics total (mean) | 12.76 SE 0.85, 95% CI 11.05-14.46 | 11.53 SE 0.65, 95% CI 10.23-12.82 | 0.2469 |
| - Motor tics | 8.24 SE 0.48, 95% CI 7.28-9.21 | 7.66 SE 0.36, 95% CI 6.94-8.38 | 0.3272 |
| - Vocal tics | 4.51 SE 0.44, 95% CI 3.62-5.4 | 3.86 SE 0.37, 95% CI 3.12-4.60 | 0.2686 |
| - Complex tics | 3.29 SE 0.47, 95% CI 2.33-4.24 | 2.82 SE 0.35, 95% CI 2.14-3.51 | 0.4222 |
| - Complex motor tics | 1.08 SE 0.14, 95% CI 0.80-1.37 | 0.89 SE 0.11, 95% CI 0.66-1.12 | 0.3006 |
| - Complex vocal tics | 2.20 SE 0.35, 95% CI 1.51- 2.90 | 1.93 SE 0.26, 95% CI 1.42- 2.44 | 0.5208 |
| - Simple tics | 9.47 SE 0.50, 95% CI 8.46-10.48 | 8.70 SE 0.41, 95% CI 7.88- 9.53 | 0.2434 |
| - Simple motor tics | 7.16 SE 0.38, 95% CI 6.40-7.93 | 6.77 SE 0.299, 95% CI 6.17- 7.37 | 0.4157 |
| - Simple vocal tics | 5.80 SE 0.67, 95% CI 4.44-7.15 | 4.68 SE 0.50, 95% CI 3.68-5.67 | 0.1748 |
| ATQ frequency total (mean) | 27.92 SE 2.47, 95% CI 22.95-32.89 | 28.04 SE 1.94, 95% CI 24.17-31.91 | 0.9688 |
| - Motor tics | 20.14 SE 1.66, 95% CI 16.80-23.48 | 20.93 SE 1.37, 95% CI 18.21-23.66 | 0.7148 |
| - Vocal tics | 7.78 SE 0.96, 95% CI 5.85-9.70 | 7.11 SE 0.79, 95% CI 5.53-8.68 | 0.5929 |
| - Complex tics | 4.47 SE 0.86, 95% CI 2.7-6.20 | 4.54 SE 0.63, 95% CI 3.28-5.80 | 0.9458 |
| - Complex motor tics | 2.49 SE 0.38, 95% CI 1.73-3.25 | 2.11 SE 0.28, 95% CI 1.55-2.66 | 0.4079 |
| - Complex vocal tics | 1.98 SE 0.53, 95% CI 0.91-3.05 | 2.43 SE 0.40, 95% CI 1.64-3.23 | 0.4905 |
| - Simple tics | 23.45 SE 1.91, 95% CI 19.61-27.28 | 23.5 SE 1.52, 95% CI 20.47-26.53 | 0.9833 |
| - Simple motor tics | 17.65 SE 1.38, 95% CI 14.88- 20.42 | 18.82 SE 1.20, 95% CI 16.43 - 21.22 | 0.5292 |
| - Simple vocal tics | 5.80 SE 0.67, 95% CI 4.44-7.15 | 4.68 SE 0.50, 95% CI 3.68-5.67 | 0.1748 |
| ATQ intensity total (mean) | 28.29 SE 2.67, 95% CI 22.91-33.66 | 27.23 SE 2.03, 95% CI 23.18-31.28 | 0.7501 |
| - Motor tics | 18.82 SE 1.55, 95% CI 15.70-21.93 | 18.15 SE 1.17, 95% CI 15.83-20.47 | 0.7269 |
| - Vocal tics | 9.47 SE 1.28, 95% CI 6.90-12.03 | 9.08 SE 1.07, 95% CI 6.94-11.22 | 0.8173 |
| - Complex tics | 2.67 SE 0.43, 95% CI 1.81- 3.53 | 2.24 SE 0.31, 95% CI 1.62-2.87 | 0.4082 |
| - Complex motor tics | 2.67 SE 0.43, 95% CI 1.81-3.53 | 2.24 SE 0.31, 95% CI 1.62-2.87 | 0.4082 |
| - Complex vocal tics | 4.88 SE 0.91, 95% CI 3.05-6.71 | 5.01 SE 0.76, 95% CI 3.49-6.53 | 0.9096 |
| - Simple tics | 20.73 SE 1.79, 95% CI 17.13-24.34 | 19.97 SE 1.28, 95% CI 17.42-22.52 | 0.7230 |
| - Simple motor tics | 16.14 SE 1.25, 95% CI 13.62-18.66 | 15.91 SE 0.98, 95% CI 13.96-17.85 | 0.8803 |
| - Simple vocal tics | 4.59 SE 0.68, 95% CI 3.23-5.95 | 4.07 SE 0.47, 95% CI 3.14-5.00 | 0.5110 |
| ATQ Total (mean) | 68.96 SE 5.73, 95% CI 57.44-80.48 | 66.80 SE 4.43, 95% CI 57.97-75.62 | 0.7634 |
| RAQ-R (mean) | 22.94 SE 2.89, 95% CI 17.14-28.74 | 19.18 SE 2.12, 95% CI 14.94-23.41 | 0.2858 |
| BAI (mean) | 14.84 SE 1.57, 95% CI 11.67-18.00 | 11.5 SE 1.19, 95% CI 9.13-13.87 | 0.0881 |
| I-8 (mean) | 5.39 SE 0.25, 95% CI 4.89- 5.88 | 4.88 SE 0.207, 95% CI 4.47-5.29 | 0.1191 |
| ADHS-SB (mean) | 2.27 SE 0.23, 95% CI 1.81-2.72 | 1.72 SE 0.178 95% CI 1.36- 2.07 | 0.0567 |
| BDI (mean) | 15.86 SE 1.58, 95% CI 12.67-19.04 | 12.30 SE 1.05, 95% CI 10.20-14.39 | 0.0534 |
| OCI (mean) | 38.63 SE 1.82, 95% CI 34.97-42.30 | 31.54 SE 1.30, 95% CI 28.95-34.133 | **0.0015** |
| BSL-23 (mean) | 20.67 SE 2.82, 95% CI 15.00-26.35 | 12.99 SE 1.44, 95% CI 10.11-15.87 | **0.0091** |
| GTS-QOL (mean) | 40.04 SE 3.08, 95% CI 33.86-46.22 | 30.88 SE 2.37, 95% CI 26.16-35.60 | **0.0185** |
| GTS VAS (mean) | 53.88 SE 3.31, 95% CI 47.22- 60.53 | 59.19 SE 2.32, 95% CI 54.58-63.80 | 0.1776 |

SE – standard error, CI – confidence interval, ADD - attention deficit disorder, ATQ – the Adult Tic Questionnaire, RAQ-R – the Rage Attack Questionnaire Revised, BAI – the Beck Anxiety Inventory, I-8 – the Impulsive Behavior Short Scale, ADHS-SB - ADHS-Selbstbeurteilungsskala, BDI – the Beck Depression Inventory, OCI – the Obsessive-Compulsive Inventory, BSL-23 – the Borderline Symptom List, GTS QOL – the Gilles de la Tourette Quality of Life Scale, GTS VAS – the Visual Analogue Scale for Quality of Life; all scales were self-assessments; statistically significant differences are noted in bold
