# Supplementary material for: Frequency of body focused repetitive behaviors and comparison to self-injurious behaviors in patients with tic disorders
Source: Sci Rep. 2025 Aug 25;15:31238. doi: 10.1038/s41598-025-12023-5 (PMC12379270; doi:10.1038/s41598-025-12023-5)
Supplement: Supplementary file 4 — Supplementary Material 4 [file 41598_2025_12023_MOESM4_ESM.docx]

Supplementary Table 4. Comparison between patients with (N=59) and without (N=64) *current* *urges* to perform body focused repetitive behaviors (BFRB), but without BFRB *behavior*.

| Variable | With BFRB urge | No urge | P value |
| --- | --- | --- | --- |
| Age (mean) | 36.15 SE 1.88, 95% CI 32.37-39.93 | 36.88 SE 1.75, 95% CI 33.38-40.37 | 0.78 |
| Sex (n,%) | 32/82, 39% (male)  27/41, 65.85% (female) | 50/82, 60.98% (male)  14/41, 34.15% (female) | **0.0047** |
| ADD (n,%) | 2/59, 3.3% | 5/64, 7.8% | 0.282 |
| ADHD (n,%) | 6/59, 10.2% | 9/64, 14.1% | 0.5 |
| Depression (n, %) | 16/59, 27.12% | 17/64, 26.6% | 0.9446 |
| Anxiety (n, %) | 11/59, 18.6% | 12/64, 18.8% | 0.988 |
| Sleeping problems (n, %) | 6/59, 10.2% | 8/64, 12.5% | 0.684 |
| ATQ (mean) number of tics total | 13.19 SE 0.75, 95% CI 11.69-14.68 | 10.94 SE 0.69, 95% CI 9.55-12.33 | **0.03** |
| - Motor tics | 8.37 SE 0.41, 95% CI 7.55-9.20 | 7.45 SE 0.40, 95% CI 6.65-8.26 | 0.113 |
| - Vocal tics | 4.81 SE 0.42, 95% CI 3.97- 5.66 | 3.48 SE 0.37, 95% CI 2.74-4.23 | **0.019** |
| - Complex tics | 3.59 SE 0.44, 95% CI 2.71-4.48 | 2.47 SE 0.34, 95% CI 1.78-3.15 | **0.044** |
| - Complex motor tics | 1.15 SE 0.136, 95% CI 0.88-1.43 | 0.8 SE 0.11, 95% CI 0.57 -1.02 | **0.046** |
| - Complex tics vocal | 2.44 SE 0.33, 95% CI 1.79 -3.10 | 1.67 SE 0.25, 95% CI 1.17-2.17 | 0.062 |
| ATQ (mean) number of simple tics total | 9.59 SE 0.437, 95% CI 8.72-10.47 | 8.46875 SE 0.460, 95% CI 7.55- 9.387 | 0.08 |
| - Motor tics | 7.22 SE 0.327, 95% CI 6.57-7.88 | 6.66 SE 0.336, 95% CI 5.99-7.33 | 0.232 |
| - Vocal tics | 7.16 SE 0.381, 95% CI 6.40 -7.93 | 6.77 SE 0.299, 95% CI 6.17-7.37 | **0.037** |
| ATQ (mean) frequency total | 31.15 SE 17.67, 95% CI 26.55-35.76 | 25.08 SE 15.67, 95% CI 21.16-28.99 | **0.046** |
| - Motor tics | 22.49 SE 1.59, 95% CI 19.32 -25.67 | 18.89 SE 1.37, 95% CI 16.15-21.63 | 0.087 |
| - Vocal tics | 8.66 SE 0.92, 95% CI 6.82-10.50 | 6.19 SE 0.78, 95% CI 4.63-7.75 | **0.0415** |
| - Complex tics | 5.36 SE 0.83, 95% CI 3.69-7.03 | 3.73 SE 0.60, 95% CI 2.54 -4.93 | 0.1122 |
| - Complex motor tics | 2.70 SE 0.36, 95% CI 1.97-3.42 | 1.86 SE 0.27, 95% CI 1.32-2.40 | 0.063 |
| - Complex vocal tics | 2.66 SE 0.53, 95% CI 1.60 -3.72 | 1.88 SE 0.37, 95% CI 1.13-2.62 | 0.221 |
| - Simple tics | 25.80 SE 1.77, 95% CI 22.25-29.34 | 21.34 SE 1.55, 95% CI 18.25-24.43 | 0.06 |
| - Simple motor tics | 19.80 SE 1.35, 95% CI 17.09-22.51 | 17.03 SE 1.20, 95% CI 14.63-19.43 | 0.128 |
| - Simple vocal tics | 6 SE 0.61, 95% CI 4.78- 7.22 | 4.31 SE 0.52, 95% CI 3.28-5.35 | **0.04** |
| ATQ (mean) intensity total | 30.41 SE 2.5, 95% CI 25.47 -35.35 | 25.11 SE 2.08, 95% CI 20.96-29.26 | 0.1012 |
| - Motor tics | 19.75 SE 1.40, 95% CI 16.94-22.55 | 17.19 SE 1.23, 95% CI 14.74-19.64 | 0.1706 |
| - Vocal tics | 10.66 SE 1.27, 95% CI 8.12-13.20 | 7.92 SE 1.033, 95% CI 5.86 -9.99 | 0.0945 |
| - Complex tics | 2.90 SE 0.43, 95% CI 2.15-3.75 | 1.92 SE 0.307, 95% CI 1.308-2.536 | **0.0424** |
| - Complex motor tics | 2.95 SE 0.401, 95% CI 2.15-3.75 | 1.92 SE 0.307, 95% CI 1.308-2.536 | **0.0424** |
| - Complex vocal tics | 5.93 SE 0.93, 95% CI 4.08-7.79 | 4.06 SE 0.71, 95% CI 2.64- 5.49 | 0.1092 |
| - Simple tics | 21.53 SE 1.61, 95% CI 18.31-24.74 | 19.125 SE 1.35, 95% CI 16.42-21.83 | 0.2531 |
| - Simple motor tics | 16.80 SE 1.15, 95% CI 14.50-19.09 | 15.27 SE 1.027, 95% CI 13.21-17.32 | 0.3208 |
| - Simple vocal tics | 4.73 SE 0.62, 95% CI 3.48-5.98 | 3.86 SE 0.48, 95% CI 2.91-4.81 | 0.2652 |
| ATQ Total (mean) | 74.75 SE 5.27, 95% CI 64.19-85.30 | 61.13 SE 4.52, 95% CI 52.09-70.16 | 0.05 |
| RAQ-R (mean) | 22.02 SE 2.56, 95% CI 16.89-27.14 | 19.44 SE 2.32, 95% CI 14.8-24.078 | 0.4558 |
| I-8 (mean) | 5.10 SE 0.243, 95% CI 4.61- 5.59 | 5.0625 SE 0.21, 95% CI 4.64- 5.48 | 0.9031 |
| BAI (mean) | 15.27 SE 1.37, 95% CI 12.52-18.02 | 10.58 SE 1.28, 95% CI 8.01-13.14 | **0.0138** |
| ADHS-SB (mean) | 2.24 SE 0.21, 95% CI 1.81-2.66 | 1.65625 SE 0.18, 95% CI 1.29-2.02 | **0.0394** |
| BDI (mean) | 15.69 SE 1.37, 95% CI 12.96-18.43 | 11.89 SE 1.16, 95% CI 9.58-14.20 | **0.0349** |
| OCI (mean) | 37.68 SE 1.65, 95% CI 34.38-40.98 | 31.31 SE 1.40, 95% CI 28.52-34.11 | **0.0037** |
| BSL-23 (mean) | 20.24 SE 2.52, 95% CI 15.20-25.27 | 12.19 SE 1.42, 95% CI 9.36- 15.02 | **0.0052** |
| GTS-QOL (mean) | 41.41 SE 2.84, 95% CI 35.72-47.09 | 28.19 SE 2.34, 95% CI 23.52 -32.86 | **0.0004** |
| GTS VAS (mean) | 51.36 SE 2.76, 95% CI 45.83-56.88 | 62.34 SE 2.53, 95% CI 57.29-67.39 | **0.0039** |

SE – standard error, CI – confidence interval, ADD - attention deficit disorder, ATQ – the Adult Tic Questionnaire, RAQ-R – the Rage Attack Questionnaire Revised, BAI – the Beck Anxiety Inventory, I-8 – the Impulsive Behavior Short Scale, ADHS-SB - ADHS-Selbstbeurteilungsskala, BDI – the Beck Depression Inventory, OCI – the Obsessive-Compulsive Inventory, BSL-23 – the Borderline Symptom List, GTS QOL – the Gilles de la Tourette Quality of Life Scale, GTS VAS – the Visual Analogue Scale for Quality of Life; all scales were self-assessments; statistically significant differences are noted in bold
