# Supplementary material for: Frequency of body focused repetitive behaviors and comparison to self-injurious behaviors in patients with tic disorders
Source: Sci Rep. 2025 Aug 25;15:31238. doi: 10.1038/s41598-025-12023-5 (PMC12379270; doi:10.1038/s41598-025-12023-5)
Supplement: Supplementary file 5 — Supplementary Material 5 [file 41598_2025_12023_MOESM5_ESM.docx]

Supplementary Table 5. Comparison between patients with both current urge and behavior related to body focused repetitive behaviors (BFRB) and those with neither BFRF urge nor BFRB behavior.

| Variable | Both BFRB urge and behavior | Neither urge nor behavior | P value |
| --- | --- | --- | --- |
| Age (mean) | 36.56 SE 1.77, 95% CI 33.03-40.09 | 36.49 SE 1.86, 95% CI 32.77-40.21 | 0.9791 |
| Sex (n,%) | 38/82, 46.3% (male)  30/41, 73.2% (female) | 44/82, 53.7% (male)  11/41, 26.8% (female) | **0.0042** |
| ADD (n,%) | 3/59, 5.1% | 4/64, 6.2% | 0.4974 |
| ADHD (n,%) | 7/59, 11.9% | 8/64, 12.5% | 0.4751 |
| OCD (n,%) | 23/59, 39% | 9/64, 14.1% | **0.0258** |
| Depression (n, %) | 16/59, 27.1% | 17/64, 26.6% | 0.3593 |
| Anxiety (n, %) | 12/59, 20.3% | 11/64, 17.2% | 0.7397 |
| Sleeping problems (n, %) | 6/59, 10.1% | 8/64, 12.5% | 0.7364 |
| ATQ number of tics total (mean) | 13.01 SE 0.69, 95% CI 11.63-14.39 | 10.78 SE 0.75, 95% CI 9.27-12.29 | **0.0313** |
| - Motor tics | 8.31 SE 0.39, 95% CI 7.53-9.1 | 7.38 SE 0.43, 95% CI 6.53-8.24 | 0.1123 |
| - Vocal tics | 4.72 SE 0.38, 95% CI 3.95-5.46 | 3.4 SE 0.41, 95% CI 2.57-4.23 | **0.0219** |
| - Complex tics | 3.53 SE 0.40, 95% CI 2.72-4.33 | 2.36 SE 0.36, 95% CI 1.63-3.09 | **0.0380** |
| - Complex motor tics | 1.13 SE 0.13, 95% CI 0.88-1.38 | 0.76 SE 0.12, 95% CI 0.52-1.007 | **0.0397** |
| - Complex vocal tics | 2.40 SE 0.30, 95% CI 1.80-2.99 | 1.6 SE 0.27, 95% CI 1.06-2.14 | 0.0540 |
| - Simple tics | 9.49 SE 0.40, 95% CI 8.68-10.29 | 8.42 SE 0.506, 95% CI 7.40-9.433287 | 0.0982 |
| - Simple motor tics | 7.18 SE 0.31, 95% CI 6.55-7.80 | 6.62 SE 0.36, 95% CI 5.91 -7.33 | 0.2395 |
| - Simple vocal tics | 2.31 SE 0.17, 95% CI 1.97- 2.65 | 1.8 SE 0.21 95% CI 1.38- 2.22 | **0.0596** |
| ATQ frequency total (mean) | 29.35 SE 17.30, 95% CI 25.17-33.54 | 26.31 SE 2.2, 95% CI 21.90-30.72 | 0.3217 |
| - Motor tics | 21.24 SE 1.46 18.32- 24.15 | 19.85 SE 1.51 16.82- 22.89 | 0.5161 |
| - Vocal tics | 8.12 SE 0.82 95% CI 6.48-9.75 | 6.45 SE 0.90 95% CI 4.65- 8.25 | 0.1746 |
| - Complex tics | 5.03 SE 0.74, 95% CI 3.55-6.51 | 3.87 SE 0.68, 95% CI 2.52-5.23 | 0.2608 |
| - Complex motor tics | 2.54 SE 0.32; 95% CI 1.90-3.19 | 1.92 SE 0.30; 95% CI 1.31-2.51 | 0.1609 |
| - Complex vocal tics | 2.49 SE 0.47; 95% CI 1.55-3.42 | 1.96 SE 0.42; 95% CI 1.11-2.81 | 0.4197 |
| - Simple tics | 24.32 SE 1.63; 95% CI 21.08-27.57 | 22.44 SE 1.73; 95% CI 18.97-25.90 | 0.4300 |
| - Simple motor tics | 18.69 SE 1.25, 95% CI 16.19-21.19 | 17.95 SE 1.32, 95% CI 15.30-20.59 | 0.6842 |
| - Simple vocal tics | 5.63 SE 0.55, 95% CI 4.54-6.72 | 4.49 SE 0.59, 95% CI 3.30-5.68 | 0.1601 |
| ATQ intensity total (mean) | 29.46 SE 2.20, 95% CI 25.06 -33.85 | 25.42 SE 2.36, 95% CI 20.70-30.14 | 0.2149 |
| - Motor tics | 19.15 SE 1.27, 95% CI 16.61-21.68 | 17.51, SE 1.37, 95% CI 14.76-20.26 | 0.3837 |
| - Vocal tics | 10.31 SE 1.12, 95% CI 8.08-12.54 | 7.92, SE 1.18, 95% CI 5.54-10.28 | 0.1454 |
| - Complex tics | 2.76 SE 0.36, 95% CI 2.05-3.48 | 1.98 SE 0.35, 95% CI 1.28-2.68 | 0.1251 |
| - Complex motor tics | 2.76 SE 0.36 95% CI 2.05-3.48 | 1.98 SE 0.35, 95% CI 1.28-2.68 | 0.1251 |
| - Complex vocal tics | 5.71 SE 0.83, 95% CI 4.06-7.36 | 4.04 SE 0.80 , 95% CI 2.43-5.64 | 0.1551 |
| - Simple tics | 20.99 SE 1.43, 95% CI 18.13-23.84 | 19.4 SE 1.53, 95% CI 16.32-22.48 | 0.4533 |
| - Simple motor tics | 16.38 SE 1.04, 95% CI 14.31- 18.45 | 15.53 SE 1.15, 95% CI 13.23- 17.83 | 0.58 |
| - Simple vocal tics | 4.60, SE 0.55, 95% CI 3.51-5.69 | 3.87, SE 0.55, 95% CI 2.78-4.97 | 0.3521 |
| ATQ Total (mean) | 71.82, SE 4.76, 95% CI 62.33-81.32 | 62.51, SE 5.11, 95% CI 52.27-72.75 | 0.1862 |
| RAQ-R (mean) | 21.32 SE 2.39, 95% CI 16.55-26.10 | 19.87 SE 2.47, 95% CI 14.91-24.83 | 0.67 |
| BAI (mean) | 14.28 SE 1.28, 95% CI 11.73-16.83 | 11.04 SE 1.42, 95% CI 8.18- 13.89 | 0.0924 |
| I-8 (mean) | 5.15 SE 0.22, 95% CI 4.70-5.60 | 5 SE 0.23, 95% CI 4.55-5.45 | 0.65 |
| ADHS-SB (mean) | 2.15 SE 0.20, 95% CI 1.75-2.54 | 1.67 SE 0.20, 95% CI 1.28- 2.07 | 0.0952 |
| BDI (mean) | 14.79 SE 1.25, 95% CI 12.29- 17.30 | 12.38 SE 1.28642 9.80-14.96 | 0.186 |
| OCI (mean) | 37.33 SE 1.49, 95% CI 34.36- 40.29 | 30.71 SE 1.54, 95% CI 27.63-33.79 | **0.0027** |
| BSL-23 (mean) | 19.10 SE 2.24, 95% CI 14.64-23.56 | 12.27 SE 1.60, 95% CI 9.07-15.47 | **0.0189** |
| GTS-QOL (mean) | 38.76 SE 2.62, 95% CI 33.54-43.99 | 29.29 SE 2.66, 95% CI 23.95- 34.63 | **0.0133** |
| GTS VAS (mean) | 53.97 SE 2.61, 95% CI 48.76-59.18 | 60.9 SE 2.79, 95% CI 55.32- 66.49 | 0.0728 |

SE – standard error, CI – confidence interval, ADD - attention deficit disorder, ATQ – the Adult Tic Questionnaire, RAQ-R – the Rage Attack Questionnaire Revised, BAI – the Beck Anxiety Inventory, I-8 – the Impulsive Behavior Short Scale, ADHS-SB - ADHS-Selbstbeurteilungsskala, BDI – the Beck Depression Inventory, OCI – the Obsessive-Compulsive Inventory, BSL-23 – the Borderline Symptom List, GTS QOL – the Gilles de la Tourette Quality of Life Scale, GTS VAS – the Visual Analogue Scale for Quality of Life; all scales were self-assessments, statistically significant differences are noted in bold
