# Supplementary material for: Frequency of body focused repetitive behaviors and comparison to self-injurious behaviors in patients with tic disorders
Source: Sci Rep. 2025 Aug 25;15:31238. doi: 10.1038/s41598-025-12023-5 (PMC12379270; doi:10.1038/s41598-025-12023-5)
Supplement: Supplementary file 6 — Supplementary Material 6 [file 41598_2025_12023_MOESM6_ESM.docx]

Supplementary Material 1.

*History of BFRB urge*, meaning that such an urge was present in the past, was reported by 56/123 patients (45%) with the following distribution: n=12/123 (9.7%) trichotillomania, n=28/123 (22.8%) skin picking, n=44/123 (35.8%), bruxism, and n=23/123 (18.7%) nail biting (multiple answers possible, for further details, please consult Supplementary Table 1).

*History of BFRB behaviors* was reported in 52/123 (42.3%) cases with n=12/123 (9.7%) reporting having history of trichotillomania, n=45/123 (36.6%) skin picking, n=39/123 (31.7%) bruxism and n=19/123 (15.44%) nail biting (multiple answers possible, for further details, please consult Supplementary Table 2).
