# Supplementary material for: Frequency of body focused repetitive behaviors and comparison to self-injurious behaviors in patients with tic disorders
Source: Sci Rep. 2025 Aug 25;15:31238. doi: 10.1038/s41598-025-12023-5 (PMC12379270; doi:10.1038/s41598-025-12023-5)
Supplement: Supplementary file 7 — Supplementary Material 7 [file 41598_2025_12023_MOESM7_ESM.docx]

Supplementary Table 6. Comparison between patients with current body focused repetitive behaviors (BFRB behaviors) and non-BFRB self-injurious behaviors (SIB behaviors) (n=71). N=37 have both BFRB and non-BFRB related SIB, N=12 pure BFRB, and N=34 pure non-BFRB related SIB.

| Variable | Non BFRB related SIB | BFRB | P value |
| --- | --- | --- | --- |
| Age (mean) | 31.91 SE 2.39, 95% CI 27.06- 36.77 | 44.00 SE 5.02, 95% CI 32.94-55.06 | **0.0196** |
| Sex (n,%) | 22/34, 64.71% (male)  12/34, 35.29% (female) | 7/12, 58.33% (male)  5/12, 41.67% (female) | 0.6956 |
| ADD (n,%) | 2/34, 5.88% | 1/12, 8.33% | 0.7730 |
| ADHD (n,%) | 6/34, 17.65% | 0/12, 0% | **0.000** |
| OCD (n,%) | 10/34, 29.41% | 1/12, 8.33% | 0.1118 |
| Depression (n, %) | 11/34, 32.35% | 4/12, 33.33% | 0.9504 |
| Anxiety (n, %) | 5/34, 14.71% | 3/12, 25% | 0.4324 |
| Sleeping problems (n, %) | 5/34, 14.71% | 2/12, 16.67% | 0.8719 |
| ATQ number of tics total (mean) | 14.35 SE 0.94, 95% CI 12.45-16.26 | 8.33 SE 1.25, 95% CI 5.58-11.08 | **0.0013** |
| - Motor tics | 8.82, SE 0.47, 95% CI 7.93-9.84 | 5.58, SE 0.93, 95% CI 3.53-7.64 | **0.0013** |
| - Vocal tics | 5.47 SE 0.58, 95% CI 4.28- 6.66 | 2.75, SE 0.45, 95% CI 1.77- 3.73 | **0.0109** |
| - Complex tics | 4.59, SE 0.55, 95% CI 3.47-5.71 | 1.08, SE 0.50, 95% CI -.016 2.18 | **0.0008** |
| - Complex motor tics | 1.41 SE 0.18, 95% CI 1.04-1.79 | 0.5 SE 0.19, 95% CI 0.07-0.93 | **0.0089** |
| - Complex vocal tics | 3.18 SE 0.41, 95% CI 2.35-4.00 | 0.58 SE 0.34, 95% CI -0.16- 1.32 | **0.0007** |
| - Simple tics | 9.76 SE 0.56, 95% CI 8.62-10.91 | 7.25 SE 0.95, 95% CI 5.17-9.33 | **0.0271** |
| - Simple motor tics | 7.47 SE 0.37, 95% CI 6.73-8.22 | 5.08 SE 0.79, 95% CI 3.34-6.83 | **0.0035** |
| - Simple vocal tics | 2.29 SE 0.28, 95% CI 1.73-2.86 | 2.17 SE 0.30, 95% CI 1.51-2.82 | 0.8011 |
| ATQ frequency total (mean) | 33.74 SE 2.95, 95% CI 27.74-39.73 | 18 SE 3.43, 95% CI 10.46- 25.54 | **0.0054** |
| - Motor tics | 24.32 SE 1.97, 95% CI 20.31-28.34 | 11.92 SE 2.29, 95% CI 6.88-16.95 | **0.0013** |
| - Vocal tics | 9.41 SE 1.23, 95% CI 6.91-11.91 | 6.08 SE 1.45, 95% CI 2.90-9.27 | 0.1461 |
| - Complex tics | 6.97 SE 1.10, 95% CI 4.74-9.20 | 1.42 SE 0.54, 95% CI 0.22-2.61 | **0.0051** |
| - Complex motor tics | 3.09 SE 0.47, 95% CI 2.13-4.05 | 1 SE 0.37, 95% CI 0.19-1.81 | **0.0156** |
| - Complex vocal tics | 3.88 SE 0.68, 95% CI 2.50 -5.27 | 0.42 SE 0.23, 95% CI 0.09- 0.92 | **0.0047** |
| - Simple tics | 26.76 SE 2.20, 95% CI 22.3-31.23 | 16.58 SE 3.08, 95% CI 9.80-23.36 | **0.0176** |
| - Simple motor tics | 21.24 SE 1.69, 95% CI 17.79-24.68 | 10.92 SE 2.03, 95% CI 6.44-15.39 | **0.0018** |
| - Simple vocal tics | 5.53, SE 0.72, 95% CI 4.07-6.99 | 5.67, SE 1.39, 95% CI 2.61-8.73 | 0.9256 |
| ATQ intensity total (mean) | 35.5 SE 3.02, 95% CI 29.35- 41.65 | 18.08, SE 3.354008 , 95% CI 10.70-25.47 | **0.0027** |
| - Motor tics | 21.85 SE 1.61, 95% CI 18.58-25.13 | 11.67, SE 2.36, 95% CI 6.47-16.87 | **0.0018** |
| - Vocal tics | 13.65 SE 1.71, 95% CI 10.18-17.12 | 6.42 SE 1.39, 95% CI 3.36-9.48 | **0.0203** |
| - Complex tics | 3.44 SE 0.54, 95% CI 2.34-4.54 | 0.67, SE 0.28, 95% CI 0.04-1.29 | **0.0047** |
| - Complex motor tics | 3.44 SE 0.54, 95% CI 2.34-4.54 | 0.67, SE 0.28, 95% CI 0.04-1.29 | **0.0047** |
| - Complex vocal tics | 8.82 SE 1.28, 95% CI 6.21-11.44 | 1.08, SE 0.61, 95% CI 0.26-2.42 | **0.0010** |
| - Simple tics | 23.24 SE 1.71, 95% CI 19.76-26.71 | 16.33, SE 3.02, 95% CI 9.68-22.99 | **0.0477** |
| - Simple motor tics | 18.41 SE 1.30, 95% CI 15.78 -21.05 | 11, SE 2.18, 95% CI 6.19-15.81 | **0.0055** |
| - Simple vocal tics | 4.82 SE 0.66, 95% CI 3.49-6.16 | 5.33, SE 1.21, 95% CI 2.67-7.99 | 0.7007 |
| ATQ Total (mean) | 83.59 SE 6.53, 95% CI 70.31-96.87 | 44.42, SE 7.70, 95% CI 27.46- 61.37 | **0.0020** |
| RAQ-R (mean) | 22.18 SE 3.46, 95% CI 15.13-29.22 | 15.5 SE 5.72, 95% CI 2.92- 28.08 | 0.3279 |
| BAI (mean) | 13.79 SE 1.89, 95% CI 11.00, 95% CI 9.95-17.63 | 11.33, SE 3.00, 95% CI 4.73-17.93 | 0.5031 |
| I-8 (mean) | 5.12 SE 0.34, 95% CI 4.43-5.80 | 5.67, SE 0.47, 95% CI 4.64-6.69 | 0.3902 |
| ADHS-SB (mean) | 2.26 SE 0.31, 95% CI 1.64-2.89 | 2 SE 0.41, 95% CI 1.10-2.90 | 0.6444 |
| BDI (mean) | 13.79 SE 1.37, 95% CI 11.02-16.57 | 10.92, SE 2.56, 95% CI 5.29-16.54 | 0.3015 |
| OCI (mean) | 35.97, SE 1.93, 95% CI 32.05-39.89 | 34.17, SE 3.72, 95% CI 25.98-42.36 | 0.6474 |
| BSL-23 (mean) | 15.35 SE 2.08, 95% CI 11.12- 19.58 | 11.42, SE 2.65, 95% CI 5.58-17.25 | 0.3119 |
| GTS-QOL (mean) | 38.71, SE 3.73, 95% CI 31.12-46.29 | 29.58, SE 4.83, 95% CI 18.96-40.21 | 0.1940 |
| GTS VAS (mean) | 58.24, SE 3.26, 95% CI 51.61-64.86 | 60 , SE 6.51, 95% CI 45.66-74.34 | 0.7933 |

SE – standard error, CI – confidence interval, ADD - attention deficit disorder, ATQ – the Adult Tic Questionnaire, RAQ-R – the Rage Attack Questionnaire Revised, BAI – the Beck Anxiety Inventory, I-8 – the Impulsive Behavior Short Scale, ADHS-SB - ADHS-Selbstbeurteilungsskala, BDI – the Beck Depression Inventory, OCI – the Obsessive-Compulsive Inventory, BSL-23 – the Borderline Symptom List, GTS QOL – the Gilles de la Tourette Quality of Life Scale, GTS VAS – the Visual Analogue Scale for Quality of Life; all scales were self-assessments, statistically significant differences are noted in bold
